# Treatment of Penetrating Gun-Shot Wounds of the Abdominal Cavity, with Report of Two Cases

**Published:** 1886-12-20

**Authors:** Jos. W. Heddens

**Affiliations:** St. Joseph, Mo.


### TREATMENT OF PENETRATING GUN-SHOT WOUNDS OF THE ABDOMINAL CAVITY, WITH REPORT OF TWO CASES.

By Jos. W. Heddens, M. D., St. Joseph, Mo.

The best method, in my opinion, in testing penetrating gun-shot wounds of the abdominal cavity, is by means of the exploratory incision. My reasons for thinking so, are these: That by this method we are enabled to make a positive diagnosis, to remove all foreign material, to arrest hemorrhage, and to lessen the danger to septicemia. It is not the size of the opening that is of importance. There is very little more danger from a large opening than from a small one. Probably the most important feature in such cases, and certainly the most frequent and certain cause of death, is hemorrhage. It is well known that hemorrhage in the peritoneal cavity is much greater from the wound of a given vessel than from a wound of a similar vessel in other regions, which fact is accounted for by the laxity of the tissue through which the vessel conrses, and the absence of atmospheric influences and pressure.

If this is true, when only a small vessel is injured, we can readily understand how, that when a large vessel is injured, the hemorrhage will prove certain and almost immediately fatal. Even if the hemorrhage is not copious enough to prove fatal of itself, it is always sufficient in amount to endanger life by secondary septic decomposition, which cannot be avoided in any other way than by abdominal section. Besides the question of hemorrhage, much more good can often be accomplished, such as removing foreign bodies, sewing up wounds, intestines, or a removal of a part, or the entirety of wounded organs.

Certain it is that the cases of rccovery by the old or do-nothing plan of treatment are very rare, when Dr. Otis, in the surgical history of the war says that the authenticated cases of recovery can be counted on the fingers of one hand. While a few cases have recovered without operative interference, this fact is not an argument against it; for by its adoption, little, if anything, is added to the serious nature of the injury, and the wounded parts are put in a much more favorable condition for recovery.

The time at which the operation should be performed is also of importance. I do not believe it is the best plan to wait for urgent symptoms to present themselves, but to operate as soon as expedient; for patients thus wounded sometimes die without urgent symptoms, and, moreover, risk would certainly be greater after urgent symptoms have arisen.

To illustrate my conviction, I will report briefly the history of two cases which have recently come under my observation, and one of which was treated by the old or expectant, and the other by the new or operative plan.

Case I.— Some months since I was called in consultation to see a Mr. T., age about thirty years, who had received a pistol-shot wound in the left inguinal region. He had no special symptoms, very little pain or tenderness, no swelling. I was of the opinion that there was hemorrhage in the abdominal cavity, and insisted upon abdominal section, but neither the attending physician or relatives would consent. He was treated on the expectant plan, and on the fifth day, while doing as well apparently as he had been at any time since receiving the wound, he suddenly died, and at the post-mortem examination it was found that the bowel was wounded, although not perforated, and one of the iliac veins perforated. He died from hemorrhage. Had the operative plan been used in this case, I am confident his chances for recovery would have been much better.

Case II.— Shortly after this I was called to see a Mr. U., age about thirty years, who had received a pistol-shot wound in the epigastric region.

He complained of great pain in the right side and back, and also in the right shoulder. He also had difficulty in breathing. I was of the opinion also that there was hemorrhage in the abdominal cavity. I explained to him and his relatives the serious nature of his injury, and also explained and urged the operative treatment Upon consultation with two other surgeons we agreed that this plan of treatment would give the patient the best chance for recovery.

We aceordingly opened the abdominal cavity, beginning the incision at the site of the wound and carrying it downwards five and one-half inches. We could trace the course of the bullet to the liver. We removed a piece of his vest from the peritoneal cavity, which had been carried in by the bullet. About four ounces of blood was also removed. The wound was dressed antiseptically, and the patient made an uninterrupted recovery, and has been in good health ever since, weighing now about two hundred pounds.

One object in reporting these cases is to call forth the expression of the members as to the best method of proceeding in such cases All of the older writers and surgeons, with very few exceptions, advocate the old or expectant plan. While this is yet a comparatively new method of treatment I am confident that it will not be long until it has an established position.—*T. Mo. M. Asso.*

